# Supplementary material for: Distinct temporal diversity profiles for nitrogen cycling genes in a hyporheic microbiome
Source: PLoS One. 2020 Jan 27;15(1):e0228165. doi: 10.1371/journal.pone.0228165 (PMC6984685; doi:10.1371/journal.pone.0228165)
Supplement: S1 Fig — Temperature (Temp), dissolved oxygen (DO), chloride ion concentration (Cl), sulfate concentration (SO4), nitrate concentration (NO3) and dissolved organic carbon (measured as non-purgeable organic carbon, NPOC) measurements were taken for all samples. Pair-wise correlation of observations were performed to determine the independence of the parameters. (PDF) [file pone.0228165.s001.pdf]

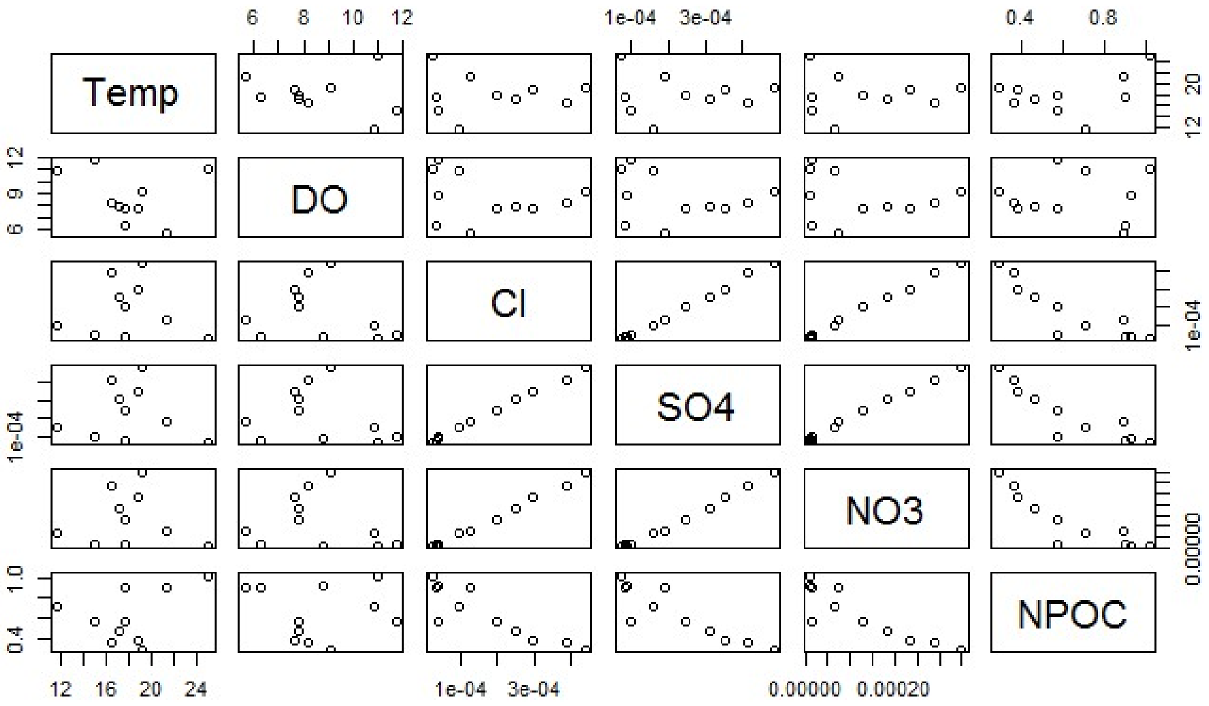

Figure S1. Environmental parameter correlation. Temperature (Temp), dissolved oxygen (DO), chloride ion concentration (Cl), sulfate concentration (SO4), nitrate concentration (NO3) and dissolved organic carbon (measured as non-purgeable organic carbon, NPOC) measurements were taken for all samples. Pair-wise correlation of observations were performed to determine the independence of the parameters.
