# Supplementary material for: Distinct temporal diversity profiles for nitrogen cycling genes in a hyporheic microbiome
Source: PLoS One. 2020 Jan 27;15(1):e0228165. doi: 10.1371/journal.pone.0228165 (PMC6984685; doi:10.1371/journal.pone.0228165)
Supplement: S4 Fig — Blue borders: p < 0.10; Orange borders: p < 0.05. (PDF) [file pone.0228165.s004.pdf]

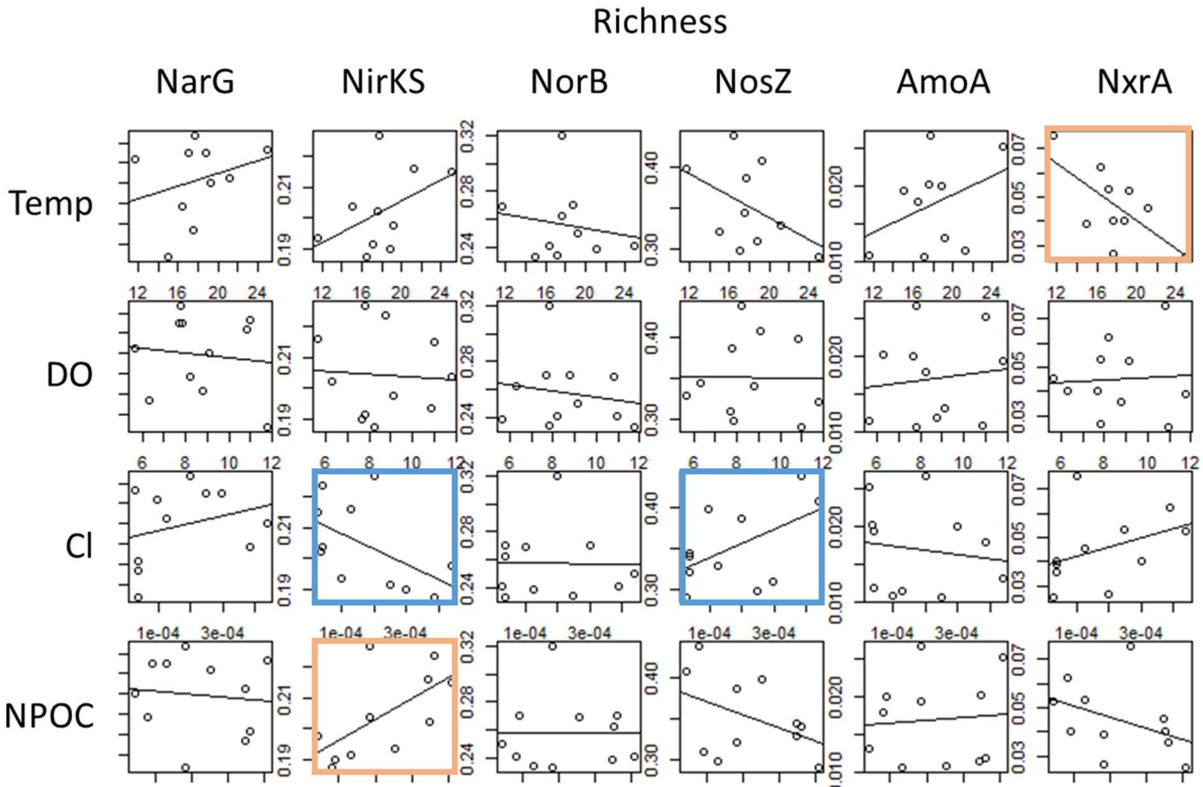

Figure S4. Environmental parameter vs richness linear regression analysis. Blue borders: $p < 0.10$; Orange borders: $p < 0.05$.
