# Supplementary material for: Distinct temporal diversity profiles for nitrogen cycling genes in a hyporheic microbiome
Source: PLoS One. 2020 Jan 27;15(1):e0228165. doi: 10.1371/journal.pone.0228165 (PMC6984685; doi:10.1371/journal.pone.0228165)
Supplement: S5 Fig — (a) Stainless steel piezometers (5.25 cm inner diameter) that were fully-screened for 1.2 m were driven into the river bottom sediment. (b) 4.5” stainless steel infusers (18/8 mesh) were packed with ~80 cm3 of locally-sourced medium grade sand (>0.425mm <1.7mm) and plugged with Pyrex fiber glass. Paired sand packs were deployed as shown in panel a) for six week incubations collected at three week intervals from April 30, 2014 to November 25, 2014. (PDF) [file pone.0228165.s005.pdf]

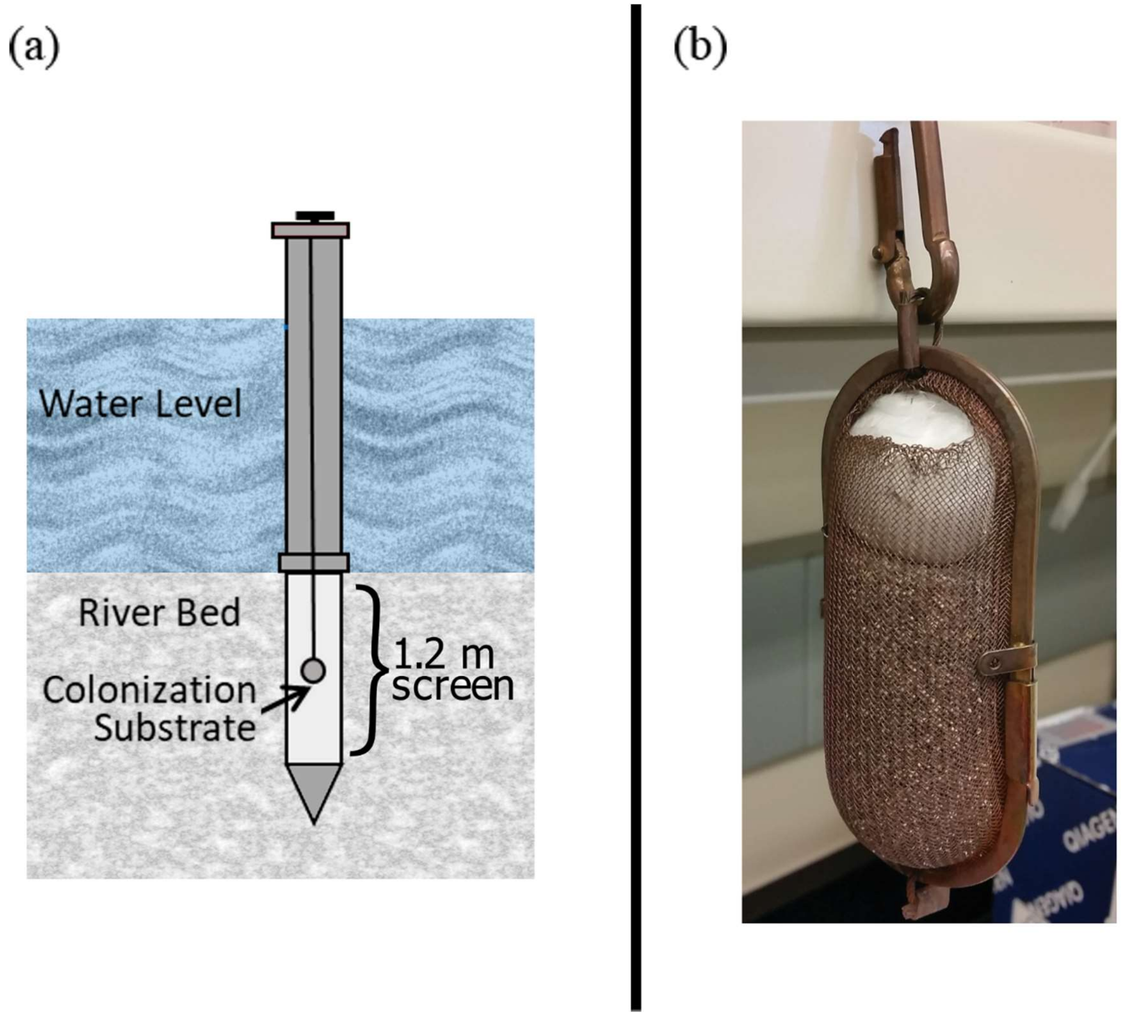

Figure S5. Sampling setup. a) Stainless steel piezometers (5.25 cm inner diameter) that were fully-screened for 1.2 m were driven into the river bottom sediment. **b)** 4.5” **s**tainless steel infusers (18/8 mesh) were packed with ~80 cm^3 of locally-sourced medium grade sand (>0.425mm <1.7mm) and plugged with Pyrex fiber glass. Paired sand packs were deployed as shown in panel a) for six week incubations collected at three week intervals from April 30, 2014 to November 25, 2014.
