# Supplementary material for: Distinct temporal diversity profiles for nitrogen cycling genes in a hyporheic microbiome
Source: PLoS One. 2020 Jan 27;15(1):e0228165. doi: 10.1371/journal.pone.0228165 (PMC6984685; doi:10.1371/journal.pone.0228165)
Supplement: S1 Table — (PDF) [file pone.0228165.s006.pdf]

Table S1 Metagenomic sequence data sets

Piezometer	Date Collected	IMG project id	Total Raw Seqs
**T4**	2014-04-30	1065983	48,412,732
**T4**	2014-05-21	1066005	25,681,518
**T4**	2014-06-10	1066001	33,554,732
**T4**	2014-07-01	1065991	19,761,996
**T4**	2014-07-22	1065977	16,159,946
**T4**	2014-08-12	1065987	19,728,928
**T4**	2014-09-02	1065979	16,770,554
**T4**	2014-09-23	1065995	36,917,050
**T4**	2014-10-14	1066003	33,326,776
**T4**	2014-11-04	1066007	62,894,882
**T4**	2014-11-25	1065981	60,562,476
