# Supplementary material for: Distinct temporal diversity profiles for nitrogen cycling genes in a hyporheic microbiome
Source: PLoS One. 2020 Jan 27;15(1):e0228165. doi: 10.1371/journal.pone.0228165 (PMC6984685; doi:10.1371/journal.pone.0228165)
Supplement: Attachment — Submitted filename: Response Letter.docx [file pone.0228165.s007.docx]

PONE-D-19-29302
Distinct temporal diversity profiles for nitrogen cycling genes in a hyporheic microbiome
PLOS ONE

Greetings, Dr. Liu.

Thank you for your diligence in guiding our manuscript through the submission process.

We are also grateful to the reviewers for volunteering their time to read and critique our work.

Our responses to editorial and reviewer comments are embedded below in red. One issue arose for which we are asking editorial guidance: our large heatmap figures contain small text that might be illegible in print versions of the manuscript. Do you think it would be best if these figures were moved to Supplemental Information to remove size restrictions, or does the electronic distribution of PLOS articles allow for magnification of figures such that this is not necessarily a detriment to the work?

**Journal Requirements:**

When submitting your revision, we need you to address these additional requirements:

Please ensure that your manuscript meets PLOS ONE's style requirements, including those for file naming. The PLOS ONE style templates can be found at

<http://www.journals.plos.org/plosone/s/file?id=wjVg/PLOSOne_formatting_sample_main_body.pdf> and <http://www.journals.plos.org/plosone/s/file?id=ba62/PLOSOne_formatting_sample_title_authors_affiliations.pdf>

**The two referenced documents have been reviewed, and the manuscript has been revised to meet the template format.**

1. Please include a copy of Table 2 which you refer to in your text on page 18

**Table 2 was mis-labeled in the initial submission. The error has been corrected.**

2. Please include captions for your Supporting Information files at the end of your manuscript, and update any in-text citations to match accordingly. Please see our Supporting Information guidelines for more information: <http://journals.plos.org/plosone/s/supporting-information>.

**The requested text has been added.**

**Review Comments to the Author**

**Reviewer #1**:

The manuscript clearly illustrated the seasonal dynamic profiles of N-cycling gene diversity in a hyporheic microbiome. It is well written and the results and discussion are presented well, except for the methods which should be described in more detail. Especially for figure 10, the analogy of an electrical circuit is very imagery and easy for readers to understand the importance of diversity in biogeochemical processes. Thus, I would suggest minor revision.
Some of specific comments:

1. Line 76, please illustrate the reason(s) of designing the three locations (T2, T3 and T4) and the differences between them.

**The three locations were spatially-separated replications sampling the hyporheic zone. This has been noted in the text.**

2. Line 105-118, in this paragraph, too many details on the determination of diversity are given in results, which is suggested to put into the methods.

**We felt that it is important for the reader to understand the data types used in the work to properly assess our analysis, thus we decided to emphasize the methods used to generate the sequences and how we define ‘phylotype’ in this work.**

3. Line 347, to better understand the sediment and aqueous sample collection, the sampling device schematic is suggested to be attached.

**A supplemental figure portraying the sampling setup has been added to the manuscript.**

4. How many samples were assigned for DNA extraction? Which DNA was assigned for sequencing (including the fraction of 16S rRNA gene and Metagenomic analysis)? And which area of 16S rRNA gene was sequenced? It is necessary to be elaborated clearly in the methods.

**T4 is the only site for which we are presenting sequence data. For each timepoint, two sandpacks were collected, combined and homogenized prior to DNA extraction. Genomic DNA preparations were divided and subjected to amplicon and shotgun sequencing. The V4 region of the 16S rRNA gene was used for amplicon analysis, as proscribed by the Earth Microbiome Project. The Methods section has been edited to include this information.**

5. In addition, gene names should be written in the formal way, such as, change 16S to 16S rRNA gene in figure 2, and write the gene names, such as narG in italic. The time ruler in the figures is recommended to be consistent.

**We feel it is fair to use abbreviations in the key for Figure 2 (now Figure 3), however, we have altered the legend text to directly associate the abbreviations used with the formal gene names. Gene names in the text have been italicized, and figures have been updated to have uniform time scale axes.**

**Reviewer #2:**

Overall a well written paper with sufficient data to support claims. I recommend the paper be published in PLOS ONE, but have some minor revision suggestions for increasing clarity to future readers. Each suggestion is listed below:

1. For all of the heat map figures (3-6), the current image quality is low and it is impossible to read the individual gene phylotype names.
   Also, in these figures some of the boxes are shaded gray - it is unclear what the gray shade means/represents, it would be great if this could be clarified in the figure legend.

**Presentation of large data sets is always challenging. We have supplied figures in the highest resolution specified by the journal. Perhaps a solution is to put larger versions of the figures in Supplemental Information. We have requested guidance from the editor on this matter.
We have stipulated in the Figure 3 (now Fig 4) legend that gray shading indicates no observation of the phylotype at that timepoint.**

2. In my opinion, I do not think the heat map figures do a good job of clearly showing changes in gene phylotype changes over time. Is it possible to graph this data another way? I was thinking an alluvial diagram would be a better way to visualize the phylotype changes. I think better graphics would increase reader accessibility.

**An alluvial diagram is an interesting idea, but in practice, our data is more numerical than categorical, and we feel that the number of phylotypes being tracked in the figures would strain the interpretability of such a figure.**

3. As currently written, it is hard for non-experts to follow the results descriptions of nitrification and denitrification genes and keep straight which genes belong to which metabolic pathway. Adding short clarifying sentences would help. For example, in line 120 it would be helpful to add a sentence about denitrification genes being discussed - similar to what is said about nitrification around line 128-129.
   If there is space, it would be useful to create a diagram that lists the steps of nitrification and denitrification in sequential order and bold/highlight each of the genes in these metabolisms you searched for during your analyses. It would be great if this diagram was in the introduction so the reader could always refer to it. This sort of graphic would help guide readers as to why it is unexpected nxrA showed little correlation with amoA (line 175-176).

**We have edited the text to give more context to the functions of the genes in question. In addition, we have added a summary figure (Figure 1) delineating the denitrification and nitrification processes.**

4. In the discussion (line 209-211, 223-225) you mention breaking the nitrification and denitrification process into component steps, yet these steps are unclear in the results, as it seems each gene is discussed individually. Adding a sentence in the results identifying which genes make up component steps and highlighting these components steps in the proposed nitrification/denitrification figure listed above would be useful for readers to follow your work.

**The component steps are simply the individual enzymatic reactions that compose the pathway, which is why each gene is discussed individually.**

5. Line 146-148 is confusing and unclear yet seemingly a critical conclusion from this work. You state that Figure 8 suggests that establishment of novel organisms, with the genes you looked at, is rare. How do you reach this conclusion? Would new unique microbes with the genes you are interested in cause an increase in diversity and a steeper curve in on the inverse Simpson plot? A brief justification would be useful.

**This figure has been expanded and the text has been edited to better relate the changes in phylotype composition for each gene from both a richness and diversity (inverse Simpson) perspective.**

6. If space, please spell out what RPKM stands for in Figure legend 9 (line 179).

**This edit has been made.**

7. A small typo exists at the end of line 215-216.

**The text has been edited.**

8. Line 405 - the HMM table needs to be table 2, and this number needs to be corrected in line 396

The text has been edited accordingly.
